# A(maize)ing attraction: gravid *Anopheles arabiensis* are attracted and oviposit in response to maize pollen odours

**DOI:** 10.1186/s12936-016-1656-0

**Published:** 2017-01-23

**Authors:** Betelehem Wondwosen, Sharon R. Hill, Göran Birgersson, Emiru Seyoum, Habte Tekie, Rickard Ignell

**Affiliations:** 1 Department of Zoological Sciences, Addis Ababa University, Box 1176, Addis Ababa, Ethiopia; 2 Unit of Chemical Ecology, Department of Plant Protection Biology, Swedish University of Agricultural Sciences, Box 102, Sundsvägen 14, 230 53 Alnarp, Sweden

**Keywords:** *Anopheles arabiensis*, Gravid mosquitoes, Maize pollen, Headspace volatiles, Olfaction, Attraction, Oviposition, Kairomone

## Abstract

**Background:**

Maize cultivation contributes to the prevalence of malaria mosquitoes and exacerbates malaria transmission in sub-Saharan Africa. The pollen from maize serves as an important larval food source for *Anopheles* mosquitoes, and females that are able to detect breeding sites where maize pollen is abundant may provide their offspring with selective advantages. *Anopheles* mosquitoes are hypothesized to locate, discriminate among, and select such sites using olfactory cues, and that synthetic volatile blends can mimic these olfactory-guided behaviours.

**Methods:**

Two-port olfactometer and two-choice oviposition assays were used to assess the attraction and oviposition preference of gravid *Anopheles arabiensis* to the headspace of the pollen from two maize cultivars (BH-660 and ZM-521). Bioactive compounds were identified using combined gas chromatography and electroantennographic detection from the headspace of the cultivar found to be most attractive (BH-660). Synthetic blends of the volatile compounds were then assessed for attraction and oviposition preference of gravid *An. arabiensis*, as above.

**Results:**

Here the collected headspace volatiles from the pollen of two maize cultivars was shown to differentially attract and stimulate oviposition in gravid *An. arabiensis*. Furthermore, a five-component synthetic maize pollen odour blend was identified, which elicited the full oviposition behavioural repertoire of the gravid mosquitoes.

**Conclusions:**

The cues identified from maize pollen provide important substrates for the development of novel control measures that modulate gravid female behaviour. Such measures are irrespective of indoor or outdoor feeding and resting patterns, thus providing a much-needed addition to the arsenal of tools that currently target indoor biting mosquitoes.

**Electronic supplementary material:**

The online version of this article (doi:10.1186/s12936-016-1656-0) contains supplementary material, which is available to authorized users.

## Background

Oviposition site selection behaviour provides a much-needed target for vector control [1], as selection of oviposition sites is an essential part of the mosquito life history, and a critical factor in their survival and population dynamics [2]. Gravid females should select enemy-free and nutrient-rich habitats for their offspring, as mosquito aquatic stages are restricted in mobility within the maternally selected habitats [2, 3]. Breeding habitats with reduced predator [4, 5] and competitor pressures [5, 6], as well as sufficient food availability, are vital for determining mosquito fitness [7], and directly affects vectorial capacity and competence [8–11]. While a number of studies have characterized predator and competitor cues, little is known about how gravid mosquitoes make use of larval nutrient cues to select breeding habitats [3]. Larval mosquito diets have hitherto been shown to contain microorganisms, including algae and bacteria, along with pollen and particulate organic detritus [6, 9, 12–14]. Maize pollen, in particular, serves as an important source of larval nourishment for *Anopheles* mosquitoes [14], which are adapted to breed in transient turbid water that can often be found associated with agricultural activities [9, 13]. Feeding on maize pollen enhances larval development, increasing the likelihood of large adults with increased longevity, fitness and resistance to insecticides [9, 11, 13, 14], and results in the intensification of malaria transmission [15]. Female mosquitoes that are able to detect breeding sites where maize pollen is abundant may thus provide their offspring with selective advantages, including survival and developmental [9, 13, 14]. Identifying and manipulating sensory cues that mediate a female mosquito’s ability to choose superior oviposition sites could provide important insights essential to developing novel mosquito control tools.

For many mosquito species, oviposition site selection is dependent on olfactory cues from plant and microbial origin [2, 3, 16]. Although some of the compounds released by these sources have been identified for *Aedes* and *Culex* mosquitoes [2, 3], we are only now starting to understand the complexity of chemical cues regulating oviposition site selection in *Anopheles* mosquitoes [16, 17]. To date, synthetic compounds that attract and stimulate oviposition in gravid *Anopheles gambiae* and *Anopheles arabiensis* have been identified from rice [17], as well as microbes associated with their larval habitats [16]. The aim of this study was to characterize the behavioural response of gravid *An. arabiensis* to volatile headspace collections from maize pollen collected from two varieties, Bako Hybrid (BH)-660 and Melkassa-2 (ZM)-521. Bako Hybrid-660 was specifically selected as a high-yield and late pollen-shedding variety, the cultivation of which has been shown to correlate with higher malaria transmission than other cultivated varieties [15]. A further aim was to show that volatiles released by pollen of both varieties elicited attraction and stimulated oviposition. The final aim was to use combined gas chromatography and electroantennographic detection analysis, together with subtractive behavioural assays, to identify a synthetic odour blend that elicits the full oviposition behavioural repertoire of gravid mosquitoes.

## Methods

### Experimental mosquitoes

*Anopheles arabiensis*, Nazareth and Dongola strains, both maintained in the laboratory for over 30 years, were used for behavioural and electrophysiological analyses in Ethiopia and Sweden, respectively. The colonies were maintained at 27 ± 2 °C, 75 ± 5% relative humidity and under a 12 h light/12 h dark cycle. Briefly, the aquatic stages of the mosquitoes were reared in distilled water, and fed Faffa food (Ethiopia) or Tetramin^®^ fish food (Sweden). Pupae were transferred from the rearing trays in 100 ml polypropylene cups (Qingdao Ori-Color Industry and Commerce Co., Ltd., China), containing distilled water, to cages (30 cm × 30 cm × 30 cm; custom-made or Bugdorm, MegaView Science, Taiwan). The emerging adults were supplied with 10% sucrose solution ad libitum. Five days after emergence, female mosquitoes were offered a rabbit (Ethiopia) or sheep blood (Sweden) from an artificial feeder (Hemotek, Discovery Workshops, Accrington, UK) over the course of two days, 3 h per day, to ensure that females took a complete blood meal. For all experiments, gravid females, 3 days post-blood feeding, were selected by visually inspecting the pale white abdomen and used for bioassays.

### Headspace volatile collection

The headspace of BH-660 and ZM-521 maize pollen was collected in the field under shaded ambient conditions at Melkassa Agricultural Research Center, in East Oromia region of Ethiopia. In addition, headspace was collected from the water of a major malaria mosquito breeding site at the shore of lake Ziway, Ethiopia. The inflorescence of a fully mature male flower (45 replicates per cultivar) was enclosed in a polyacetate bag (Toppits, Cofresco, Germany), and a charcoal-filtered continuous airstream (1 l min^−1^) was drawn by a Personal Air Sampler (PAS-500, Spectrex, Redwood City, CA, USA) over the tassel, onto an aeration column for 3 h. Alternatively, for collecting the headspace from breeding water (35 replicates), 1 l was poured into a polyacetate bag, after which the headspace was collected for 3 h using a diaphragm vacuum pump (12 V, KNF-Neuberger, Freiburg, Germany), using charcoal-filtered air as described above. The aeration columns were made of Teflon tubing (6 cm × 3 mm i.d.), filled with 35 mg Super Q (80/100 mesh; Alltech, Deerfield, IL, USA) between polypropylene wool plugs and nylon stoppers. The aeration columns were cleaned with 1 ml n-hexane (LabScan, Malmö, Sweden), re-distilled before use. Adsorbed volatiles were eluted with 300 µl re-distilled n-hexane. Headspace volatile extracts were stored in glass vials at −20 °C until used for behavioural and electrophysiological analyses.

### Two-port olfactometer

A two-port olfactometer [17] was used to test the attraction preference of the mosquitoes for the headspace volatiles collected from the BH-660 and ZM-521 pollen, and from natural breeding water. All assays were conducted between 18:00 and 21:00, which is the peak period of oviposition activity as determined in pilot experiments. For each experiment, 10 gravid females were allowed to acclimatize for 5 min in a custom-made cage (22 cm × 30 cm × 12 cm; L:W:H) constructed of clear vinyl for easy viewing. Thereafter, two dental-wick (4 cm × 1 cm; L:D; DAB Dental AB, Upplands Väsby, Sweden) odour dispensers were simultaneously introduced into the cylindrical vinyl arms (13 cm × 9 cm; L:D) positioned at opposite ends of the cage. Treatment and control wicks were exchanged in between experiments to assure for no positional bias. The ends of the cylindrical arms were covered by mesh. Attraction preference of the mosquitoes to the following treatments were analysed: (a) headspace volatiles of breeding water vs hexane control, (b) headspace volatiles of BH-660 or ZM-521 pollen vs hexane, (c) headspace volatiles of BH-660 or ZM-521 pollen vs headspace volatiles of breeding water, and (d) headspace volatiles of BH-660 vs headspace volatiles ZM-521 pollen. For all treatments, a hexane vs hexane control was performed. The behavioural response to the volatile headspace of the two maize pollen varieties was analysed to increasing amounts of headspace extract from the two pollen varieties. After 5 min, the behavioural responses of the mosquitoes were scored by counting the number of mosquitoes in each arm. Ten replicates per treatment and per release rate were performed. The treatments were tested one release rate at a time, with a solvent only control for each replicate to control for day effects. Between each replicate, the bioassay apparatus was cleaned with 70% ethanol and the position of the treatments changed to avoid bias.

### Oviposition bioassay

The oviposition preference of gravid mosquitoes was analysed in a two-choice assay [17]. Metal wire framed cages (30 cm × 30 cm × 30 cm) covered with white nylon mosquito netting were used. Two 100 ml polypropylene cups (Qingdao, Ori-Color Industry and Commerce, Co. Ltd, China), placed in opposite corners of the cages and filled to the brim (100 ml) with distilled or field collected breeding water, served as the oviposition substrate. The position of the cups was exchanged between experiments. Treatment cups were conditioned by dosing the oviposition substrate with the same range of release rates of headspace volatile extracts of BH-660 and ZM-521 pollen in hexane, as described above. An equivalent amount of hexane was used as a control. In addition, a hexane vs hexane control was performed for all treatments. Treatment and control cups were exchanged in between experiments to assure for no positional bias. Gravid mosquitoes were transferred from the maintenance cages at dusk (18:00), and on the following day (09:00) the numbers of eggs in the two cups were counted. All experiments were replicated ten times. The treatments were tested one release rate at a time, with a solvent only control for each replicate to control for day effects.

### Electrophysiological analysis

Antennal responses of gravid female *An. arabiensis* to the preferred headspace extract of BH-660 pollen were analysed using combined gas chromatography and electroantennographic detection (GC-EAD). An Agilent Technologies 6890 GC (Santa Clara, CA, USA) was equipped with a HP-5 column (30 m × 0.25 mm i.d., 0.25 μm film thickness, Agilent Technologies), and hydrogen was used as the mobile phase at an average linear flow rate of 45 cm s^−1^. Each sample (2 µl) was injected in splitless mode (30 s, injector temperature 225 °C). The GC oven temperature was programmed from 35 °C (3 min hold) at 10 °C min^−1^ to 290 °C (10 min hold). At the GC effluent, 4 psi of nitrogen was added and split 1:1 in a Gerstel 3D/2 low dead volume four-way cross (Gerstel, Mülheim, Germany) between the flame ionization detector and the EAD. The GC effluent capillary for the EAD passed through a Gerstel olfactory detection port-2 transfer line, that tracked the GC oven temperature, into a glass tube (30 cm × 8 mm), where it was mixed with charcoal-filtered, humidified air (1.5 l min^−1^). The antenna was placed 0.5 cm from the outlet of this tube. The antennal preparation was made by using the excised head. The distal end of the antenna was inserted into a recording glass electrode filled with Beadle–Ephrussi Ringer, after cutting the distal segment. The recording electrode was connected to a pre-amplifier probe and then to a high impedance DC amplifier interface box (IDAC-2; Syntech, Kirchgarten, Germany). The reference electrode, filled with Beadle–Ephrussi Ringer, was inserted into the head capsule. Each individual animal accounted for a single replicate, and at least five replicates were performed.

### Chemical analysis

The BH-660 pollen headspace extract was injected (2 µl) and analysed on a combined gas chromatograph and mass spectrometer (GC–MS; 6890 GC and 5975 MS; Agilent Technologies), operated in the electron impact ionization mode at 70 eV. The GC was equipped with fused silica capillary columns (30 m × 0.25 mm, 0.25 µm film thickness), DB-wax (J&W Scientific, Folsom, CA, USA) or HP-5MS (Agilent Technologies). Helium was used as the mobile phase at an average linear flow rate of 35 cm s^−1^. The temperature programmes were the same as for the GC-EAD analysis. Compounds were identified according to retention times (Kovat’s indices) and mass spectra, in comparison with custom made and NIST05 libraries (Agilent), and confirmed by co-injection of authentic standards: (±)-*α*-pinene (Cas no. 7785-70-8; Aldrich, 98%), (±)-limonene (Cas no. 5989-27-5; Sigma, 97%), nonanal (Cas no. 124-19-6; Aldrich, 95%), benzaldehyde (Cas no. 100-52-7; Aldrich, 99%), *p*-cymene (Cas no. 99-87-6; Aldrich, 97%). For quantification, 100 ng of heptyl acetate (99.8% chemical purity; Aldrich) was added as an internal standard.

### Bioassay with synthetic blend

The assays were carried out in the same two-port olfactometer and oviposition bioassay that were used for the natural headspace extract experiments. The synthetic blend mimicked the composition and ratio of compounds in the headspace collected from BH-660 pollen. Synthetic blends were prepared at seven different doses in half orders of magnitude between 1 and 1000 ng of α-pinene in pentane dispensed from dental-wicks and in distilled water for the attraction and oviposition assays, respectively. The ratio among the compounds within the blend was maintained as a constant across all doses. Thereafter, subtractive blends, in which single compounds of the full blend were removed, were tested against the full blend (100 ng).

### Statistical analysis

A preference index was calculated, (T − C)/(T + C), for both attraction preference (AP) and oviposition (OP) preference; where T is the number of mosquitoes or eggs associated with the test odours, and C the number of mosquitoes or eggs associated with the control odours. The behavioural responses of gravid *An. arabiensis* in the two-port olfactometer and oviposition bioassay were analysed using a nominal logistic fit model, in which choice was the dependent variable, weighted by the number of (1) mosquitoes in the attraction assays and (2) eggs laid in the oviposition assays, with release rate or dose as the independent fixed effect and replicate (day) as a random effect (JMP^®^ Pro 12.0.1. SAS Institute Inc., Cary, NC, USA). Here, we report the χ^2^ and *P* value from the likelihood ratio test. The generation of the good fit logistic models were made by omitting the highest release rate or dose, which in most cases was shown to cause either a neutral or avoidance behaviour.

## Results

### Maize pollen volatiles attract gravid mosquitoes

**Fig. 1:**
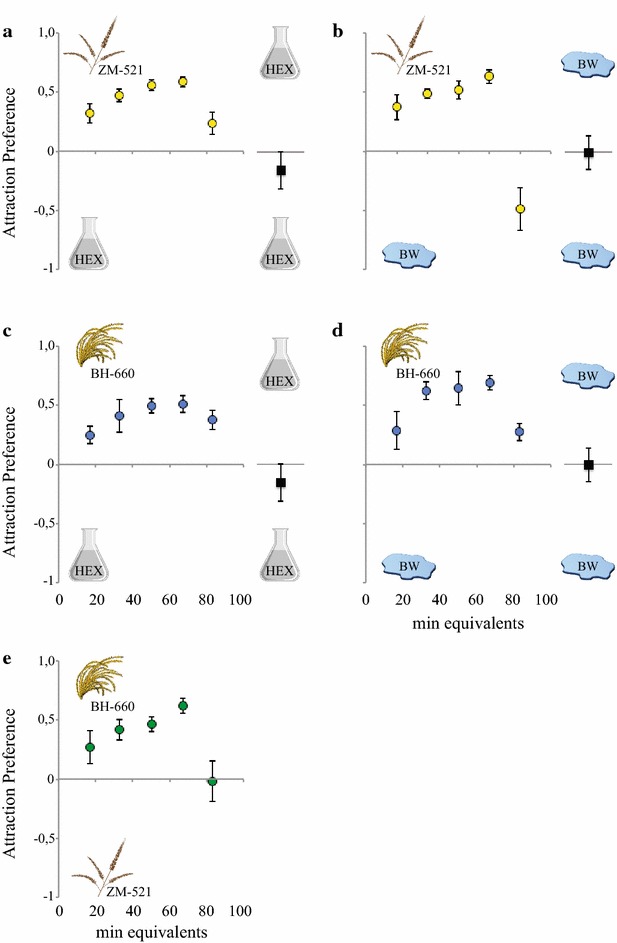
Headspace volatiles of ZM-521 and BH-660 maize pollen attract gravid *Anopheles arabiensis*. Attraction preference of mosquitoes to headspace volatiles of the ZM-521 (**a, b**) and BH-660 (**c, d**) maize pollen compared to the controls, hexane (H, *left*) and headspace of breeding water (BW, *right*), in the two-port olfactometer. The headspace of the BH-660 maize pollen was significantly preferred over that of the ZM-521 (**e**). Controls (hexane vs hexane) are shown next to the dose response analyses as *black squares* (**a**–**d**). An attraction index of zero indicates preference to neither treatment nor control. *Error bars* denote standard error of the mean

Gravid *An. arabiensis* were significantly attracted, over a range of release rates, to the headspace collections of pollen collected from the ZM-521 and BH-660 maize varieties in a two-port olfactometer, when compared to the hexane control (ZM-521: χ^2^ = 9.647, *P* = 0.0019; BH-660: χ^2^ = 8.976, *P* = 0.0027; Fig. 1a, c; Additional file 1) and the headspace of breeding water (ZM-521: χ^2^ = 10.66, *P* = 0.0011; BH-660: χ^2^ = 16.77, *P* < 0.0001; Fig. 1b, d; Additional file 1). Comparison between the two varieties showed a significantly higher attraction to the headspace of BH-660 pollen by gravid *An. arabiensis* than to that of ZM-521 (χ^2^ = 9.648, *P* = 0.0019; Fig. 1e; Additional file 1). No significant difference was observed in the attraction to the hexane control and the headspace of breeding water when tested in the same assay (χ^2^ = 0.5968, *P* = 0.4398).

### Maize pollen volatiles stimulate oviposition in gravid mosquitoes

**Fig. 2:**
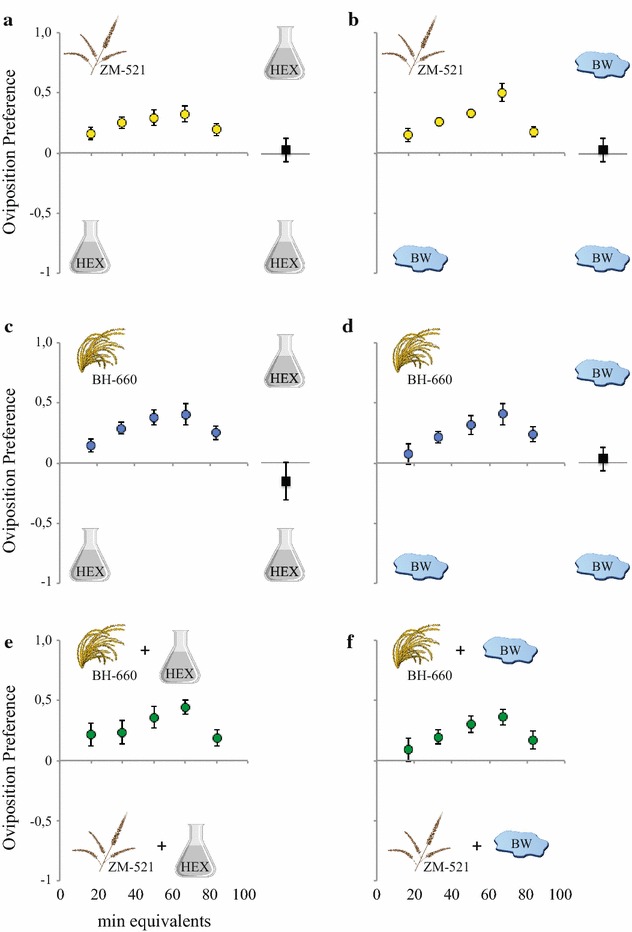
Headspace volatiles of BH-660 and ZM-521 maize pollen stimulate oviposition by gravid *Anopheles arabiensis*. Oviposition preference of mosquitoes to headspace volatiles of the ZM-521 (**a, b**) and BH-660 (**c, d**) maize pollen compared to controls, distilled (*left*) and breeding (*right*) water, conditioned with hexane. In the two-choice oviposition assay, the headspace of the BH-660 maize pollen was significantly preferred over that of the ZM-521 in both distilled (**e**) and breeding (**f**) water. Controls (hexane vs hexane) are shown next to the dose response analyses as *black squares* (**a**–**d**). An oviposition index of zero indicates preference to neither treatment nor control. *Error bars* denote standard error of the mean

Gravid females preferred to lay their eggs in both distilled and breeding water conditioned with the headspace of ZM-51 and BH-660 maize pollen, over water with hexane added as a control (ZM-521 vs distilled water: χ^2^ = 4.405, *P* = 0.0358; BH-660 vs distilled water: χ^2^ = 7.887, *P* = 0.0050; ZM-521 vs breeding water: χ^2^ = 8.980, *P* = 0.0027; BH-660 vs breeding water: χ^2^ = 6.812, *P* = 0.0091; Fig. 2a–d; Additional file 2). Comparison between the headspace of the two varieties demonstrated a significantly higher number of eggs laid by *An. arabiensis* in response to the headspace of BH-660 pollen than to that of ZM-521, irrespective of the oviposition substrate (distilled water: χ^2^ = 11.71, *P* = 0.0006; Fig. 2e; breeding water: χ^2^ = 8.492, *P* = 0.0036; Fig. 2f; Additional file 2). The number of eggs laid by *An. arabiensis* was not significantly different between the two controls (χ^2^ = 0.1959, *P* = 0.6581; data not shown).

### Identification of bioactive compounds in maize pollen headspace

**Fig. 3:**
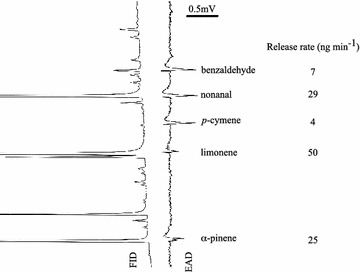
Antennal responses of gravid *Anopheles arabiensis* to BH-660 pollen volatile compounds. Electroantennographic detection (EAD) trace depicts voltage changes (mV) in response to the bioactive compounds in the headspace of BH-660 pollen eluting from the gas chromatograph and detected by the flame ionization detector (FID). The identity and release rate (ng min^−1^) of the bioactive compounds are shown at the right

The GC-EAD and GC–MS analyses identified five bioactive compounds in the headspace of the more attractive BH-660 pollen: benzaldehyde, nonanal, *p*-cymene, limonene and *α*-pinene (Fig. 3). The overall volatile release rate was 100 ng min^−1^, with limonene as the most abundant compound, followed by nonanal and *α*-pinene.

### Behavioural response to synthetic maize pollen odour

**Fig. 4:**
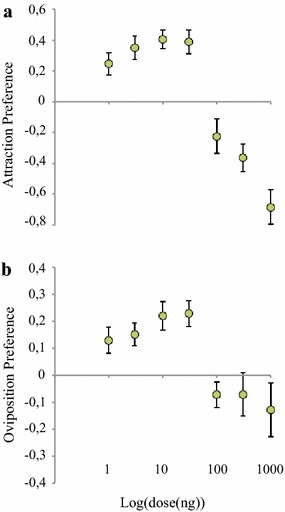
Synthetic maize pollen blend attracts and stimulates oviposition in gravid *Anopheles arabiensis*. Attraction (**a**) and oviposition (**b**) preference of gravid mosquitoes to the full five component synthetic blend presented at various doses. An attraction or oviposition index of zero indicates preference to neither treatment nor control. *Error bars* denote standard error of the mean

**Fig. 5:**
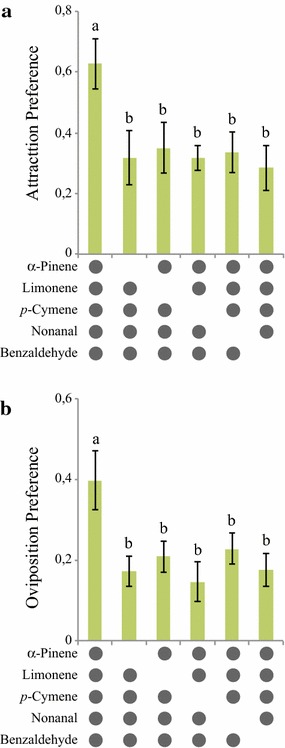
Behavioural responses of gravid *Anopheles arabiensis* to subtractive blends of compounds from BH-660 maize pollen. Attraction (**a**) and oviposition (**b**) preference of gravid mosquitoes to the subtractive blends were significantly reduced to that of the full blend (nominal logistic regression). Different *lowercase letters* indicate significant differences by odd ratios (likelihood ratio test) pairwise comparisons. *Error bars* denote standard error of the mean

A synthetic blend containing all five GC-EAD-active compounds, approximating their natural ratio (10:5:5:1:1, limonene:nonanal:*α*-pinene:benzaldehyde:*p*-cymene), elicited short-range attraction and stimulated oviposition in gravid *An. arabiensis* over a range of doses (χ^2^ = 9.581, *P* = 0.0020; χ^2^ = 8.125, *P* = 0.0044, respectively) (Fig. 4; Additional file 3). The release rate that elicited the optimal behavioural responses was found to be similar to the natural release rate (Fig. 4). To assess the role of each individual component in the blend, five subtractive blends, from which single compounds were removed, were evaluated against the full blend in both behavioural assays (Fig. 5; Additional file 4). All subtractive blends were found to be less attractive (χ^2^ = 4.02, *P* < 0.0449) and females laid fewer eggs in the subtractive blends (χ^2^ = 5.13, *P* < 0.0236) than in the full blend (Fig. 5; Additional file 4).

## Discussion

Intensification of maize cultivation in sub-Saharan Africa contributes to the propagation of vector mosquitoes, and the expansion or exacerbation of malaria transmission [15]. One key link in this interaction is the presence of maize pollen in the breeding habitats of *Anopheles* mosquitoes, created by existing irrigation schemes [9, 15]. Here, gravid *An. arabiensis* were shown to be attracted to volatiles released by maize pollen, and identified a synthetic maize pollen odour blend that attracts and stimulates oviposition. The identification of habitat odour cues from oviposition sites, preferentially selected by *Anopheles* mosquitoes, provides important substrates for future mosquito control measures that target gravid malaria mosquitoes.

The five-component blend identified from BH-660 maize pollen constitutes the second synthetic blend identified from a cereal crop [17] shown to attract and stimulate oviposition in gravid *An. arabiensis*. Three components, limonene, *α*-pinene and nonanal, are shared between the synthetic maize pollen blend and the eight-component blend identified from the headspace of the MR3 rice cultivar [17]. For both blends, the full component blends are required to elicit the full behavioural repertoire of the gravid mosquitoes, which is in line with studies on herbivorous insects showing that blend perception is critical for host plant recognition and behavioural responses [18].

Although the odour space of mosquitoes [19, 20] has yet to be definitively defined, it is interesting that volatile compounds identified as oviposition cues, also appear as salient features of other essential resources used during the gonotrophic cycle of the female. Of the five blend components identified in this study, limonene, *α*-pinene and benzaldehyde are among the most common floral odour constituents [21] and are known to attract nectar-seeking mosquitoes [21–25]. The other two components of the blend, *ρ*-cymene and nonanal, are less abundant floral constituents [21, 26] however, the behavioural significance of these compounds as floral attractants for mosquitoes is yet to be revealed [22, 26]. Benzaldehyde, *α*-pinene and nonanal have also been shown to be present in human odour headspace [27, 28] and have been shown to play a role in host attraction of mosquitoes [29–32]. As the perception of odour blends changes with the physiological state of the female [33, 34] it is likely that the importance of individual compounds will depend on the context in which they are detected.

For the selection of oviposition site by mosquitoes, the ten components identified from cereal crops, along with the assumed soil associated microbial component, cedrol [16] are the only known oviposition attractants for *Anopheles* mosquitoes. Considering the generally non-overlapping oviposition habitat choices of *Anopheles, Aedes* and *Culex* mosquitoes [2, 12] it is interesting that *Aedes* and *Culex* mosquitoes generally are attracted to a different subset of volatile compounds than *Anopheles* [3, 35, 36]. This separation of odour space and habitat choice may be related to the inherent competition between sympatric species.

The ability to interrupt the oviposition site selection behaviour of malaria mosquitoes provides needed additional target to be exploited in the development of novel control methods. Combined with existing intervention strategies (indoor residual spray and long-lasting insecticide treated nets), odour baited gravid traps could help alleviate the increasing problem of outdoor malaria transmission.

## Conclusions

In this study, gravid malaria mosquitoes were shown to be attracted to an affordable volatile synthetic blend, providing concrete evidence of a substrate that can be used as a lure in a gravid trap. Ongoing semi-field and field trials are aimed at validating the efficacy of the synthetic lure. At the same time, chimeric blends, based on the bioactive volatile compounds identified in other cereal grasses, are under evaluation.

## Additional files

**Additional file 1.** Number of individual gravid *Anopheles arabiensis* responding in the attraction assay to headspace volatile extracts of BH-660 and ZM-521 maize cultivars.

**Additional file 2.** Number of eggs laid by gravid *An* *opheles arabiensis* in the oviposition assay in response to headspace volatile extracts of BH-660 and ZM-521 maize cultivars.

**Additional file 3.** Number of individual gravid *Anopheles arabiensis* attracted and eggs laid in the oviposition assay in response to the full synthetic blend.

**Additional file 4.** Number of individual gravid *Anopheles arabiensis* attracted and eggs laid in the oviposition assay in response to the synthetic blends.

## Abbreviations

AP: attraction preference
BH: Bako hybrid
C: control
EAD: electroantennogram detection
GC: gas chromatography
GLM: general linear model
HSD: honestly significance difference
MS: mass spectrometry
OP: oviposition preference
T: test
ZM: Melkassa-2
