# Supplementary material for: A(maize)ing attraction: gravid Anopheles arabiensis are attracted and oviposit in response to maize pollen odours
Source: Malar J. 2017 Jan 23;16:39. doi: 10.1186/s12936-016-1656-0 (PMC5259891; doi:10.1186/s12936-016-1656-0)
Supplement: Supplementary file 1 — Additional file 1. Number of individual gravid Anopheles arabiensis responding in the attraction assay to headspace volatile extracts of BH-660 and ZM-521 maize cultivars. [file 12936_2016_1656_MOESM1_ESM.docx]

**Additional file 1: Number of individual gravid *Anopheles arabiensis* responding in the attraction assay to headspace volatile extracts of BH-660 and ZM-521 maize cultivars**

| **Figure** | **Dose** | **Control** | **Test** |
| --- | --- | --- | --- |
| Fig. 1a | (min equivalents) | Hexane | Extract of ZM-521 pollen |
|  | 0 | 13 | 14 |
|  | 16 | 26 | 46 |
|  | 32 | 18 | 48 |
|  | 48 | 16 | 56 |
|  | 64 | 16 | 62 |
|  | 80 | 31 | 50 |
| Fig. 1b |  | Extract of breeding water | Extract of ZM-521 pollen |
|  | 0 | 14 | 16 |
|  | 16 | 20 | 40 |
|  | 32 | 17 | 48 |
|  | 48 | 18 | 53 |
|  | 64 | 13 | 55 |
|  | 80 | 28 | 17 |
| Fig. 1c |  | Hexane | Extract of BH-660 pollen |
|  | 0 | 13 | 14 |
|  | 16 | 26 | 41 |
|  | 32 | 13 | 36 |
|  | 48 | 19 | 54 |
|  | 64 | 16 | 53 |
|  | 80 | 22 | 46 |
| Fig. 1d |  | Extract of breeding water | Extract of BH-660 pollen |
|  | 0 | 14 | 16 |
|  | 16 | 22 | 38 |
|  | 32 | 13 | 54 |
|  | 48 | 14 | 60 |
|  | 64 | 12 | 58 |
|  | 80 | 25 | 48 |
| Fig. 1e |  | Extract of BH-660 pollen | Extract of ZM-521 pollen |
|  | 16 | 38 | 23 |
|  | 32 | 47 | 21 |
|  | 48 | 56 | 21 |
|  | 64 | 61 | 15 |
|  | 80 | 22 | 32 |
