# Supplementary material for: A(maize)ing attraction: gravid Anopheles arabiensis are attracted and oviposit in response to maize pollen odours
Source: Malar J. 2017 Jan 23;16:39. doi: 10.1186/s12936-016-1656-0 (PMC5259891; doi:10.1186/s12936-016-1656-0)
Supplement: Supplementary file 2 — Additional file 2. Number of eggs laid by gravid An opheles arabiensis in the oviposition assay in response to headspace volatile extracts of BH-660 and ZM-521 maize cultivars. [file 12936_2016_1656_MOESM2_ESM.docx]

**Additional file 2: Number of eggs laid by gravid *Anopheles arabiensis* in the oviposition assay in response to headspace volatile extracts of BH-660 and ZM-521 maize cultivars**

| **Figure** | **Dose** | **Control** | **Test** |
| --- | --- | --- | --- |
| Fig. 2a | (min equivalents) | Hexane | Extract of ZM-521 pollen |
|  | 0 | 1449 | 1601 |
|  | 16 | 1677 | 2250 |
|  | 32 | 1262 | 2100 |
|  | 48 | 1284 | 2289 |
|  | 64 | 1376 | 2600 |
|  | 80 | 1564 | 2365 |
| Fig. 2b |  | Extract of breeding water | Extract of ZM-521 pollen |
|  | 0 | 1394 | 1261 |
|  | 16 | 1565 | 1181 |
|  | 32 | 2068 | 1231 |
|  | 48 | 2781 | 1407 |
|  | 64 | 2510 | 844 |
|  | 80 | 2456 | 1743 |
| Fig. 2c |  | Hexane | Extract of BH-660 pollen |
|  | 0 | 1449 | 1601 |
|  | 16 | 1614 | 2111 |
|  | 32 | 1359 | 2527 |
|  | 48 | 1163 | 2549 |
|  | 64 | 1080 | 2405 |
|  | 80 | 1314 | 2157 |
| Fig. 2d |  | Extract of breeding water | Extract of BH-660 pollen |
|  | 0 | 1394 | 1261 |
|  | 16 | 1293 | 1156 |
|  | 32 | 2249 | 1426 |
|  | 48 | 2247 | 1361 |
|  | 64 | 2457 | 941 |
|  | 80 | 2102 | 1329 |
| Fig. 2e |  | Extract of BH-660 pollen in hexane | Extract of ZM-521 pollen in hexane |
|  | 16 | 1880 | 1076 |
|  | 32 | 2533 | 968 |
|  | 48 | 2946 | 867 |
|  | 64 | 3535 | 862 |
|  | 80 | 2605 | 1484 |
| Fig. 2f |  | Extract of BH-660 pollen in breeding water | Extract of ZM-521 pollen in breeding water |
|  | 16 | 2339 | 1574 |
|  | 32 | 2666 | 1424 |
|  | 48 | 2935 | 1209 |
|  | 64 | 3379 | 1229 |
|  | 80 | 2445 | 1418 |
