# Supplementary material for: A(maize)ing attraction: gravid Anopheles arabiensis are attracted and oviposit in response to maize pollen odours
Source: Malar J. 2017 Jan 23;16:39. doi: 10.1186/s12936-016-1656-0 (PMC5259891; doi:10.1186/s12936-016-1656-0)
Supplement: Supplementary file 3 — Additional file 3. Number of individual gravid Anopheles arabiensis attracted and eggs laid in the oviposition assay in response to the full synthetic blend. [file 12936_2016_1656_MOESM3_ESM.docx]

**Additional file 3: Number of individual gravid *Anopheles arabiensis* attracted and eggs laid in the oviposition assay in response to the full synthetic blend.**

| **Figure** | **Dose** | **Control** | **Test** |
| --- | --- | --- | --- |
|  | **Attraction** | | |
| Fig. 4a |  | Full synthetic blend | Pentane |
|  | 0.1 | 34 | 21 |
|  | 0.3 | 41 | 20 |
|  | 1 | 40 | 17 |
|  | 3 | 54 | 25 |
|  | 10 | 18 | 31 |
|  | 30 | 14 | 27 |
|  | 100 | 7 | 29 |
|  | **Oviposition** | | |
| Fig. 4b |  | Full synthetic blend | Pentane |
|  | 0.1 | 5199 | 4027 |
|  | 0.3 | 5573 | 4151 |
|  | 1 | 5917 | 3814 |
|  | 3 | 6617 | 4273 |
|  | 10 | 4632 | 5299 |
|  | 30 | 4333 | 4884 |
|  | 100 | 4100 | 5262 |
