# Supplementary material for: A(maize)ing attraction: gravid Anopheles arabiensis are attracted and oviposit in response to maize pollen odours
Source: Malar J. 2017 Jan 23;16:39. doi: 10.1186/s12936-016-1656-0 (PMC5259891; doi:10.1186/s12936-016-1656-0)
Supplement: Supplementary file 4 — Additional file 4. Number of individual gravid Anopheles arabiensis attracted and eggs laid in the oviposition assay in response to the synthetic blends. [file 12936_2016_1656_MOESM4_ESM.docx]

**Additional file 4:** **Number of individual gravid *Anopheles arabiensis* attracted and eggs laid in the oviposition assay in response to the synthetic blends**

| **Figure** | **Synthetic blend** | **Control (Pentane)** | **Test (synthetic blend)** |
| --- | --- | --- | --- |
|  | **Attraction** | | |
| Fig. 5a | Full blend | 13 | 50 |
|  | Reduced α-pinene | 18 | 35 |
|  | Reduced limonene | 15 | 31 |
|  | Reduced p-cymene | 16 | 31 |
|  | Reduced nonanal | 16 | 32 |
|  | Reduced benzaldehyde | 21 | 38 |
|  | **Oviposition response** | | |
| Fig. 5b | Full blend | 2466 | 5768 |
|  | Reduced α-pinene | 3382 | 4759 |
|  | Reduced limonene | 3265 | 5049 |
|  | Reduced p-cymene | 3640 | 4899 |
|  | Reduced nonanal | 3037 | 4581 |
|  | Reduced benzaldehyde | 3459 | 4922 |
